# Endothelial progenitor cells inhibit platelet function in a P-selectin-dependent manner

**DOI:** 10.1186/s12967-015-0508-y

**Published:** 2015-05-07

**Authors:** Haissam Abou-Saleh, Ahmed Hachem, Daniel Yacoub, Marc-Antoine Gillis, Yahye Merhi

**Affiliations:** Qatar Cardiovascular Research Center, Qatar Foundation, Education City, Doha, Qatar; Laboratory of Thrombosis and Hemostasis, Montreal Heart Institute, 5000 Belanger, Montreal, H1T 1C8 QC Canada; Laboratoire d’Immunologie Cellulaire et Moléculaire, Centre de recherche du Centre Hospitalier de l’Université de Montréal, Montreal, QC Canada; Faculty of Medicine, Université de Montréal, Montreal, QC Canada

**Keywords:** Endothelial progenitor cells, Platelets, P-selectin, Aggregation, Thrombosis

## Abstract

**Background:**

The role of endothelial progenitor cells (EPCs) in vascular repair is related to their recruitment at the sites of injury and their interaction with different components of the circulatory system. We have previously shown that EPCs bind and inhibit platelet function and impair thrombus formation via prostacyclin secretion, but the role of EPC binding to platelet P-selectin in this process has not been fully characterized. In the present study, we assessed the impact of EPCs on thrombus formation and we addressed the implication of P-selectin in this process.

**Methods:**

EPCs were generated from human peripheral blood mononuclear cells cultured on fibronectin in conditioned media. The impact of EPCs on platelet aggregation and thrombus formation was investigated in P-selectin deficient (P-sel^−/−^) mice and their wild-type (WT) counterparts.

**Results:**

EPCs significantly and dose-dependently impaired collagen-induced whole blood platelet aggregation in WT mice, whereas no effects were observed in P-sel^−/−^ mice. Moreover, in a ferric chloride-induced arterial thrombosis model, infusion of EPCs significantly reduced thrombus formation in WT, but not in P-sel^−/−^ mice. Furthermore, the relative mass of thrombi generated in EPC-treated P-sel^−/−^ mice were significantly larger than those in EPC-treated WT mice, and the number of EPCs recruited within the thrombi and along the arterial wall was reduced in P-sel^−/−^ mice as compared to WT mice.

**Conclusion:**

This study shows that EPCs impair platelet aggregation and reduce thrombus formation *via* a cellular mechanism involving binding to platelet P-selectin. These findings add new insights into the role of EPC-platelet interactions in the regulation of thrombotic events during vascular repair.

## Background

Endothelial progenitor cells (EPCs) play a pivotal role in vascular biology and homeostasis, as they enhance the process of re-endothelialisation and neo-vascularization of injured and ischemic tissues [1-5]. Interactions of EPCs with vascular and blood cells can largely influence their biological activity and impact their recruitment to target tissues. More specifically, interactions of EPCs with circulating platelets enhance their functional properties and provide the critical signal to ensure their migration and homing at the sites of vascular injury, thus facilitating their differentiation into endothelial cells [6-12]. Indeed, platelets favor the adhesion of EPCs onto the sub-endothelial matrix, as they constitute a bridging mechanism for the firm arrest of EPCs on collagen surfaces under dynamic flow *in vitro* and on the injured vessel wall *in vivo* [9,13]. This adhesive interaction between EPCs and platelets may improve EPC function and induce the secretion of various vasoactive substances that can modulate the micro-environment of the lesion, which in turn may enhance vascular repair and accelerate the healing process.

Platelet aggregation represents the onset of thrombus formation during endothelial disruption. At the sites of vascular injury, platelets roll and interact with various components of the sub-endothelial matrix *via* a number of adhesive receptors expressed on the platelet surface [14]. This leads to the adhesion and activation of platelets, which is accompanied by the translocation and exposure of P-selectin (CD62P) on the membrane. P-selectin, a member of the selectin family of adhesion molecules, is expressed primarily on activated platelets [15] and promotes the interaction of platelets with leucocytes [16]. In this regard, it has been shown that P-selectin is also involved in the adhesive interaction between platelets and EPCs, an interaction which reportedly dictates their functions during vascular repair [6-9,13,17]. Indeed, previous studies have shown that specific inhibition of P-selectin reduced the accumulation of EPCs on the surface of collagen-adherent platelets in parallel-plate flow chambers and at sites of endothelial disruption [9,13].

The functional impact of EPC/platelet interactions was indirectly explored in a number of studies showing that EPCs participate in the prevention of hybrid graft thrombosis and rejection and late stent thrombosis [18-24]. Moreover, in a rat model of chronic thrombosis, transplanted EPCs appear to alter the vein micro-environment by up-regulating cytokines associated with thrombi re-organization and recanalization [25]. Taken together, this strongly suggests that EPCs may play a potential role in the management of thrombotic reactions. However, the direct effect of EPCs on the formation of platelet aggregates, presumably a critical biological event during the initial phase of thrombus formation, has not been fully explored. Nevertheless, in our previous work, we showed that human EPCs regulate platelet function, *via* up-regulation of cyclooxygenase (COX)-2 and prostacyclin (PGI_2_)-dependent inhibition of platelet activation, aggregation, adhesion, and thrombus formation [26]. Given that platelet P-selectin is the major receptor involved in the interaction of EPCs with platelets [6,8,9], we accordingly speculated that the impact of EPCs on platelet function occurs through a mechanism that involves platelet P-selectin. We, therefore, designed this study to depict the role of P-selectin in this process by assessing platelet aggregation and thrombus formation in wild type (WT) and P-selectin deficient (P-sel^−/−^) mice. We demonstrate that P-selectin is a determinant adhesive receptor involved in the recruitment of EPCs into the platelet thrombi and along the injured vessel wall. Moreover, we found that P-selectin plays a key role in mediating the inhibitory effect of EPCs on platelet aggregation *in vitro* and thrombus formation *in vivo*. This study adds new insight into the interplay between EPCs and platelets in the maintenance of vascular hemostasis and the management of thrombotic reactions.

## Methods

### Mice

Female 12 to 14 week-old C57BL/6 P-sel^−/−^ and their counterparts C57BL/6 WT mice were purchased from Jackson Laboratory (Bar Harbor, ME). This study was carried out in strict accordance with the recommendations in the guide for the care and use of laboratory animals of the Canadian council for animal care. The protocol was approved by the committee on the ethics of animal experiments of the Montreal heart institute. All surgery was performed under a mixture of ketamine (Vetalar: 1.5 mg/kg I.P., Bioniche, Belleville, ON) and medetomidine (Domitor: 1 mg/kg I.P., Pfizer, Kirkland, QC).

### Culture and characterization of human EPCs

Human EPCs were generated from peripheral blood mononuclear cells (PBMCs) collected from the anticubetal vein and cultured for 10 days on fibronectin in the presence of EndoCult^TM^ medium, as previously described [26]. Changes in cell morphology during the differentiation process were assessed by optical microscopy, whereas changes in the expression of cell surface markers were assessed by flow cytometry, as previously described [26]. This study was approved by the ethical committee of the Montreal heart institute. All human subjects were healthy volunteers of either sex, aged between 20 and 55 years old. They gave written informed consent prior to participating in the study and were free from any drugs that interfere with platelet function.

### Platelet aggregation

Blood was drawn by cardiac puncture, from anesthetized mice with a mixture of 75 mg/kg of Ketamine (Vetalar, Belleville, QC) and 0.5 mg/kg of medetomidine (Domitor, Pfizer , Kirkland, QC), in 1-cc syringes containing 50 μL of heparin (100 IU/mL) [27,28]. Blood was then subjected to the aggregation process in a 4 channel whole blood platelet aggregometer (Chrono-log corp., Havertown, PA), as previously described [28-31]. Briefly, blood was diluted 1:1 with 0.4 mL of saline solution. Fresh culture media (200 μL) or EPCs (125 × 10^3^ to 500 × 10^3^ cells/200 μL) were added to the sample of diluted blood, and allowed to warm to 37°C for 5 minutes. Platelet aggregation was induced by collagen (3 μg/mL, Chrono-log corp.) at 37°C with a stirring speed of 1000 rpm, and was expressed as the change in electrical impedance (ohms) after 5 minutes of aggregation time.

### Mouse carotid thrombosis

The effects of EPCs (125 × 10^3^ to 500 × 10^3^ cells) on thrombus formation were determined in a ferrous chloride (FeCl_3_)-mouse carotid injury model, according to a standardized protocol [26,32], and as described in our previous work [26]. Control experiments were done with fresh culture media alone. Briefly, P-sel^−/−^ and WT mice were anesthetized and the right carotid artery was carefully exposed. A miniature ultrasound flow probe (0.5 VB 552; Transonic Systems, Ithaca, NY), interfaced with a flow meter (T206; Transonic Systems) was positioned around the artery, and blood flow was analyzed through a computer-based data acquisition program (Iox 2.2.17.19, Emka, Falls Church, VA). After measuring baseline blood flow, a 0.5 × 1.0-mm strip of filter paper (Whatman no. 1) soaked in 6.5% FeCl_3_ was applied on the adventitial surface proximal to the flow probe for 3 min, after which blood flow and time to thrombotic occlusion (blood flow of 0 mL/min) were monitored.

### Histology, immune-staining and confocal fluorescence

After completion of *in vivo* blood flow measurements in thrombosis mice model, the injured and contralateral non-injured carotids were excised and fixed in 10% buffered Formalin (Starplex Scientific Inc., Etobicoke, ON). These arterial segments were then embedded in paraffin, sectioned at 6 microns, and stained with hematoxylin and eosin, or an anti-CD34 antibody (Santa Cruz). Sections were visualized using an Olympus BX60 microscope (Olympus imaging America Inc., Center Valley, PA) and the computerized morphometric analyses were performed using a Retiga 2000R camera (QImaging Corporation, Surrey, BC), and Image Pro Plus 6.2 software (Media Cybernetics, Bethesda, MD).

For confocal fluorescence, 500 × 10^3^ EPCs were labeled with an intracellular fluorescent marker (CellTracker^TM^ CM-DiI, Molecular Probes) according to the manufacturer’s instructions. Non-labeled PBMCs were used as negative control. Cells were then washed with PBS, resuspended in fresh media, injected intravenously, and allowed to circulate for 15 minutes before mouse carotid injury induction. The injured as well as the contralateral non-injured carotids were excised immediately after completion of blood flow measurements and immersed in liquid nitrogen. Labeled EPCs incorporated into the luminal aspect of arterial thrombi were observed on cryostat sections of 14-μm thickness using confocal microscopy [26].

### Statistical analysis

All data are presented as mean ± SEM of at least 4 independent experiments. Statistical comparisons were done using a one-way ANOVA, followed by Benferroni multiple comparisons test. Data with *P* <0.05 were considered statistically significant.

## Results

### Phenotypic characterization of EPCs

**Figure 1:**
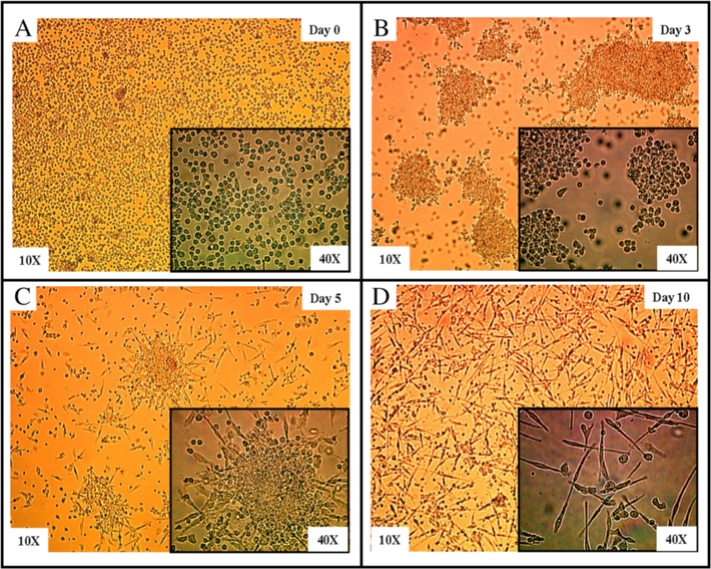
Morphological change of PBMCs-derived EPCs *in vitro.* Adherence, sequential changes and differentiation of PBMCs are observed under inverted optical microscopy. **A)** At day 0, freshly isolated PBMCs were plated on fibronectin and the majority of cells are non-adherent with a rounded morphology. **B)** At day 3, the adherent cells appear either as single cells or as irregular colony-like structures. **C)** At day 5, colonies are better defined and form a central cluster of round cells with elongated spindle-like cells at the periphery. **D)** At day 10, cells show a flat monolayer of spindle-shaped cells.

We followed the differentiation of PBMCs into EPCs *in vitro* using optical microscopy. As shown in Figure 1, freshly isolated PBMCs at day 0 appeared as single cells with rounded morphology. Cultured in conditioned media, PBMCs started to assemble into colony-like structures with irregular size after 3 days of culture. At day 5, the colonies became more organized and consisted of a central cluster of round cells and sprouts of elongated cells at the periphery, a characteristic of the colony forming units-endothelial cells. After 10 days, the cells formed a monolayer of spindle-shaped flat cells characteristic of EPCs.

**Figure 2:**
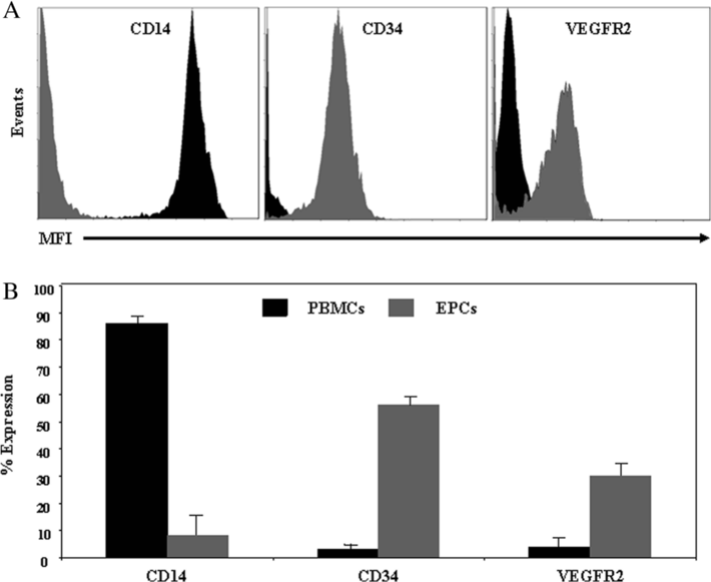
Expression of cell surface markers. **A)** Representative overlay plots showing the expression of cell surface markers, as determined by flow cytometry. Single color immunostaining of freshly isolated PBMCs at day 0 (black plots) and culture-derived EPCs at day 10 (gray plots) was performed with saturating concentrations of mouse anti-human PE-conjugated monoclonal antibodies directed against CD14, CD34 and VEGFR2. Overlay plots are presented as the number of events over the log of associated fluorescence. **B)** Histogram represents the mean data ± SEM of at least 4 independent experiments.

To further characterize the differentiation of PBMCs into EPCs, we employed flow cytometry to quantify the expression of typical markers on the surface of the cells during their differentiation (Figure 2). Cells were gated with respect to CD14/CD34 expression. We observed that freshly isolated PBMCs highly expressed the pan-leukocyte markers CD14 (86% ± 2%), whereas the progenitor/endothelial markers CD34 and vascular endothelial growth factor receptor 2 (VEGFR2) were missing (Figure 2). After 10 days of culture, progenitor/endothelial markers were expressed (56% ± 2% CD34 and 30% ± 5% VEGFR2), whereas the leukocyte marker CD14 was absent (Figure 2). Based on these characteristics, we used EPCs after 10 days of culture in the following experiments.

### EPCs inhibited platelet aggregation and thrombus formation in a P-selectin-dependent manner

**Figure 3:**
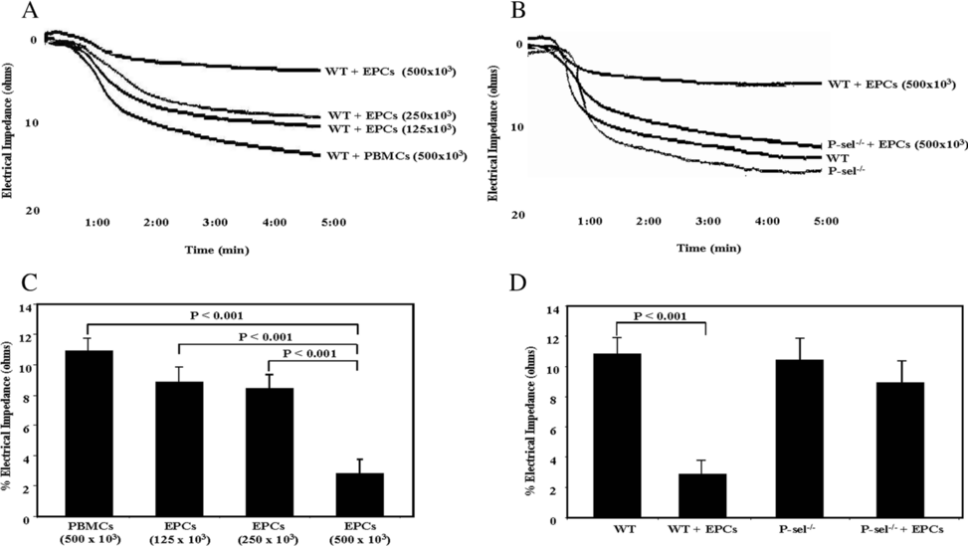
Effect of EPCs on collagen-induced platelet aggregation. Whole blood was pre-incubated with different concentrations of EPCs in a 4-channel lumi-aggregometer under shear (1,000 rpm) at 37°C. Platelet aggregation was initiated by adding collagen (3 μg/mL) and then monitored for 5 minutes. Representative traces of whole blood platelet aggregation from **A)** WT and **B)** P-sel^−/−^ mice. The mean data ± SEM of 5 independent experiments, summarizing the effects of different EPC concentrations on collagen-induced platelet aggregation in WT and P-sel^−/−^ mice are presented in **C** and **D**, respectively.

The effect of EPCs on platelet function was first assessed *in vitro* by aggregation assays. Addition of EPCs to whole blood from WT mice inhibited collagen-induced platelet aggregation in a concentration-dependent manner, ranging from 22% inhibition with 125 x 10^3^ EPCs to 75% inhibition with 500 x 10^3^ EPCs (Figure 3A and B). In contrast, EPCs were unable to significantly affect aggregation of platelets from P-sel^−/−^ mice (Figure 3C and D).

**Figure 4:**
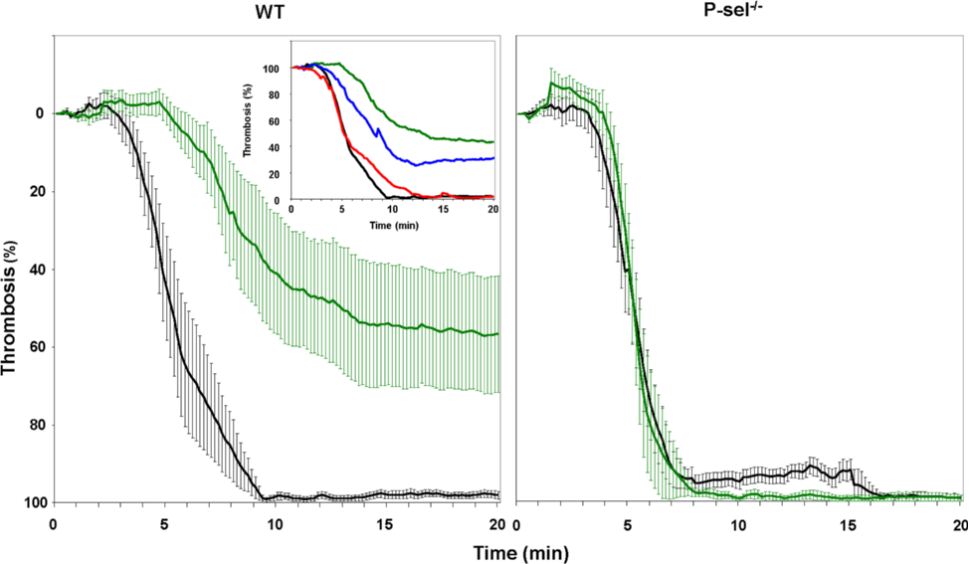
Effect of EPCs on thrombus formation. Fresh culture media or EPCs were infused and allowed to circulate for 5 minutes, followed by application of FeCl_3_ for 3 minutes and continuously monitoring of carotid blood flow for 20 minutes post-FeCl_3_ injury. Thrombus formation in WT and P-sel^−/−^ mice infused with fresh culture media (Black line, n = 7) or with 500 x 10^3^ cells/mouse (Green line, n = 7). Insert: Effects of increasing concentrations of EPCs ranging from 125 x 10^3^ cells/mouse (red line, n = 7), 250 x 10^3^ cells/mouse (blue line, n = 7), or 500 x 10^3^ cells/mouse (green line, n = 7) in WT mice.

Having shown the importance of platelet P-selectin in this process, we sought to determine its role in *in vivo* thrombus formation using a murine model of carotid thrombosis. Intravenous injection of increasing concentrations of EPCs in WT mice resulted in an increment of arterial blood flow and prolongation of time to occlusion in a concentration-dependent manner (Figure 4). Similarly to platelet aggregation, the highest rates of blood flow were reached with a concentration of 500 × 10^3^ EPCs/mouse. In marked contrast, these effects were not observable in P-sel^−/−^ mice, in which injection of 500 × 10^3^ EPCs induced similar blood flow measurements to control treated mice that received fresh culture media (Figure 4).

**Figure 5:**
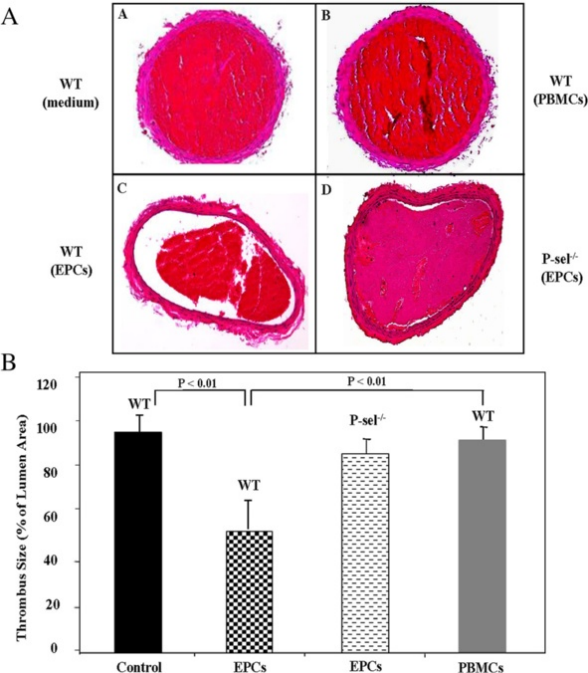
Histological cross-sections of FeCl_3_-injured mouse carotid arteries. **A)** Representative histological transverse sections of FeCl_3_-injured mouse carotid arteries treated with EPCs, PBMCs, or fresh culture media (control) and stained with hematoxylin and eosin. (Magnifications 20X). Arterial thrombus mass was completely occlusive in control-and PBMC-treated mice (panels A & B, n = 4) and partially occlusive in arteries from EPC-treated mice (panel C, n = 4). Panel D represent the injured artery from P-sel^−/−^ mice treated with 500 x 10^3^ EPCs (n = 4). **B)** Histogram represents the mean data ± SEM of cross-sectional area of arterial thrombi expressed as percentage of lumen area.

To further analyze the characteristics of the formed thrombus, injured carotid arteries from WT and P-sel^−/−^ mice were fixed and subjected to histological and morphometrical analysis. Carotids from culture media-treated mice were used as negative control. The circumferences of the arteries and thrombi were measured by computer-assisted planimetry, and the thrombus size was reported as percentage of total lumen area. In comparison to control- and PBMC-treated mice, in which the thrombus was completely occlusive (Figure 5A, panels A and B), the thrombus size in EPC-treated mice was visually decreased (Figure 5A, panel C). Indeed, injection of 500 × 10^3^ EPCs into WT mice lead to a 47% reduction in thrombus mass (Figure 5A panel C and Figure 5B). In contrast, P-sel^−/−^ mice were unresponsive to this treatment, as EPCs failed to induce significant effects and the thrombus formed was almost occlusive (Figure 5A, panel D and Figure 5B).

Hence, these results indicate that the presence of P-selectin is of utmost importance, first to promote the interaction between EPCs and platelets, and second to sustain their mechanism of action on platelet aggregation and thrombus formation.

### EPCs are recruited to the sites of injury through P-selectin

**Figure 6:**
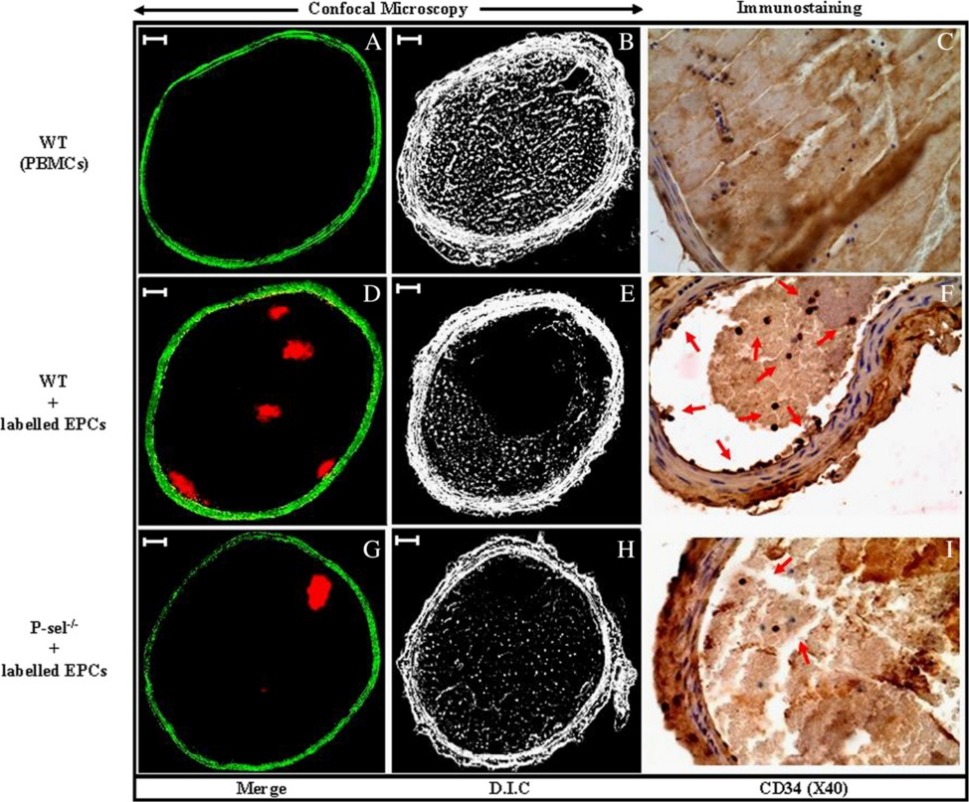
Fluorescent confocal imaging and immunostaining of FeCl_3_-injured mouse carotid arteries. Photomicrographs show fluorescent confocal imaging and immunostaining of mouse carotid arteries after vascular injury. EPCs were labeled with an intracellular fluorescence marker (DiL) 1 hour prior to injection in WT (Panel **D**) and P-sel^−/−^ (Panel **G**) mice and assessed by confocal fluorescence on cryostat cross-sections. Panel **A** represent the injured arteries from non-labeled PBMC-treated mice used as negative control. The corresponding Differential Interference Contrast (D.I.C) of the identical sections of each carotid is shown on the right (panels **B**, **E **& **H**). (Scale bar = 50 μm). Anti-CD34 immunostaining of the injured arteries in EPC-treated mice show that CD34 positive cells were uniformly distributed within the thrombus and along the vessel wall in WT - but poorly distributed in P-sel^−/−^ mice confirming the recruitment of EPCs to the luminal aspect of arterial thrombi *in vivo* (arrows in **F** and **I**). The cells in **C** are CD34 negative. The images are representative of at least 4 independent experiments.

The role of P-selectin on EPCs’ impairment of thrombus formation was further depicted by assessing their recruitment into the sites of injury. FeCl_3_-injured carotid arteries from EPC- and PBMC-treated mice (WT and P-sel^−/−^) were immediately fixed following blood flow measurements and subjected to fluorescence confocal and immunohistochemical analysis. EPCs were labeled with a photostable fluorescent cell tracker prior to intravenous injection. Labeled EPCs were identified in the luminal aspect of the thrombi and appeared as a cluster of red fluorescent clumps in the carotids of WT mice (Figure 6D), and to a lesser extent in those of P-sel^−/−^ mice (Figure 6G). Injured arteries from unlabelled PBMC-treated mice, used as negative control, were void from any staining (Figure 6A). In accordance with morphometrical analysis, the mass of thrombi generated in PBMC-treated mice showed a complete occlusion (Figure 6B), whereas thrombi mass was partially occlusive in WT mice treated with 500 × 10^3^ EPCs (Figure 6E). As expected, thrombi mass was majorly occlusive in P-sel^−/−^ mice treated with 500 x 10^3^ EPCs (Figure 6H).In another set of experiments, cross-histological sections of injured arteries from PBMC- and EPC-treated mice were immunostained with an anti-CD34 antibody. CD34 positive cells were uniformly distributed within the thrombus and along the vascular wall of EPC-injected WT mice (Figure 6F), but poorly distributed in mice from the P-sel^−/−^ group (Figure 6I). The carotids of PBMC-treated mice (negative control) showed no staining (Figure 6C).

All together, these data confirm that, in addition to its role in mediating EPC/platelet interactions, P-selectin promotes and sustains the anti-thrombotic effects of EPCs.

## Discussion

The present study provides novel insights into the biology of EPCs and their interaction with activated platelets. Specifically, we assessed the impact of EPCs on platelet aggregation and thrombus formation and we highlighted the role of platelet P-selectin in this process. We found that EPCs inhibit platelet aggregation *in vitro*, incorporate into the formed thrombi and along the vessel walls, and reduce thrombus extent *in vivo*. Moreover, we provide evidences that the inhibitory action of EPCs on platelet aggregation and thrombus formation is largely P-selectin dependent.

Arterial thrombosis with its clinical cardiovascular complications is a multi-step process that is initiated by the formation of a platelet aggregate at sites of vessel injury. Depending on the mass of the generated thrombus, partial or complete obstruction of blood flow may occur within the injured artery, which can lead to acute coronary syndromes and myocardial infarction. This process is governed by specific cell adhesion molecules which induce homotypic and heterotypic binding between platelets and other blood cells [33,34]. Among these, P-selectin has emerged as an important cell-cell interaction mediator which contributes, among others, to the recruitment of blood leukocytes [16] and circulating EPCs [6-9,13,17]. In addition to its role in thrombosis and inflammation, P-selectin has been shown to induce migration, homing and differentiation of EPCs at the sites of vascular lesion [6-10].

The role of EPCs in the maintenance of vascular integrity and as "repair" cells in response to endothelial injury is well recognized. Indeed, EPCs circulate within the blood as "surveillance" cells encountering only transitory contacts with the intact vascular endothelium. When vessel damage occurs, EPCs are sued to engage, *via* autocrine and paracrine mechanisms, in the normal response to injury. Requirement of vascular repair is communicated to circulating EPCs by adherent/aggregated platelets at sites of vessel damage.

The use of *ex vivo*-expanded EPCs has been shown to improve re-endothelialisation and neovascularization of ischemic hind limbs and ischemic hearts in animal models, as well as in the prevention of hybrid graft thrombosis, restenosis and rejection [18-24,35,36]. In particular, in a rat model of chronic thrombosis, transplanted EPCs appear to alter the vein micro-environment by up-regulating cytokines associated with thrombus re-organization and recanalization [25]. Moreover, recent clinical studies have provided ample evidence that the use of EPC-capture stents conceivably limits the cascade of events leading to acute or sub-acute stent- and late-stent thrombosis [22,23]. Taken together, these results highlight the potential role that EPCs may play in the management of thrombotic reactions. However, the direct effect of EPCs on platelet aggregation, a critical biological event during the initial phase of thrombus formation, has not been extensively studied. Nevertheless, we have previously demonstrated that EPCs bind and inhibit platelet aggregation *in vitro* and reduce thrombus formation *in vivo* [26]. However, the therapeutic effects of EPCs in cell therapy may stem from factors other than the cells themselves, as very low numbers are incorporated into capillaries post ischemia [37-39]. Instead, this important phenomenon may rather be related to their ability to release pro-angiogenic factors. In accordance with this hypothesis, we and others have previously shown that EPCs release various vasoactive substances in the micro-environment of the injury including angiogenic and growth factors, vascular endothelial growth factor, hepatocyte growth factor, stromal-derived factor, granulocyte colony-stimulating factor, insulin-like growth factor as well as PGI_2_ and nitric oxide (NO) [9,10,21,25,26,40-43]. In fact, PGI_2_ and NO are well known thrombo-resistant factors that are released from EPCs in large quantities in response to various stimuli [21]. However, the release of anti-platelet mediators near the damaged endothelium can enhance angiogenesis, inhibit platelet aggregation and limit thrombogenesis [26,44,45]. In our pervious study, we have shown that both EPCs and their supernatants were able to inhibit platelet aggregation and thrombus formation. Analysis of EPC supernatants revealed the presence of anti-thrombotic substances released by EPCs in culture such as PGI_2_ and NO. Moreover, the incubation of cultured EPCs with different PGI_2_ and NO inhibitors revealed that the inhibition of platelet function is predominantly PGI_2_-dependent. In tissues, PGI_2_ acts in paracrine manner, has a very short half-life and is rapidly metabolized into 6-keto-PGF_1α_, which is a weaker platelet inhibitor. In the present study, we speculate that the adhesive interaction between EPCs and platelets, *via* P-selectin, may create a biochemical microenvironment that favors the secretion of PGI_2_ by EPCs and optimize its biological activity on bound platelets at the site of vascular injury and thrombus formation. This may explain in part our observation that a physical link between EPCs and platelets is needed to achieve optimal inhibitory effects on thrombosis.

In this regard, it has been shown that P-selectin can mediate the rolling of platelets on activated endothelium and various cell types including EPCs. These adhesive interactions may enhance EPC activity and induce the generation of intracellular signals leading to the secretion of various vasoactive and anti-thrombotic substances that can modulate the micro-environment of the lesion and alter thrombus formation. However, this issue needs to be further addressed in detail to fully validate this intriguing possibility.

In the present study, we have shown that EPCs impair platelet aggregation and thrombus formation through P-selectin-dependent EPC/platelet interactions. Our findings are in accordance with studies from other groups showing that blockade or lack of P-selectin drastically reduces the accumulation of EPCs on the surface of adherent platelets and at the sites of endothelial denudation [9,13]. Accordingly, we have adequately succeeded to demonstrate that in deficient mice, the lack of P-selectin has substantially compromised the adhesive interaction between EPCs and platelets and consequently reversed the inhibitory effect of EPCs on platelet aggregation *in vitro* and thrombus formation *in vivo*. The importance of EPC/platelet interactions in vascular biology was further validated by our cell recruitment data, which show that EPCs are recruited to the thrombus mass in a process that depends upon P-selectin. These findings may be of important physiological relevance in vascular homeostasis since EPCs must integrate into blood vessels and platelet thrombi to improve neovascularization and limit thrombogenesis. However, our data leaves open which ligand on EPCs is necessary for platelet P-selectin mediated interaction. This is due to the heterogeneity of EPC populations found in the circulation or differentiated in culture and to the diversity of cell adhesion molecules expressed on their surfaces. Nevertheless, we showed that PBMC-derived EPCs bind activated platelets in a P-selectin-dependent manner, thus confirming previous findings demonstrating that the interaction between platelets and EPCs occurs, in part, via P-selectin and its high affinity receptor PSGL-1. However, this does not rule out the involvement of other mediators such as SDF-1α, CXC chemokine receptor-2 and −4, and β_1_- and β_2_-integrins, which may also participate in EPC binding to platelets and homing at the sites of vascular injury [6-10,13,17,46,47].

## Conclusions

Endothelial progenitor cells represent a promising therapeutic approach for the treatment of cardiovascular diseases. Interaction of EPCs with platelets is critical for their recruitment and the fulfillment of their potential therapeutic properties during vascular repair. In this study, we provided novel insights into the central role of P-selectin in the recruitment and interaction of EPCs with platelets at the sites of vascular injury mainly within the thrombi and along the vessel wall. In addition, we demonstrated that EPCs inhibit platelet aggregation and thrombus formation through a mechanism that involves the engagement of P-selectin; thus highlighting the therapeutic potential of EPCs, which may be relevant to the management of atherothrombosis during acute coronary syndromes and following percutaneous coronary interventions. In addition to the well-documented roles of EPCs in angiogenesis and vascular repair, our findings highlight a new biological role for EPCs in regulating platelet function via P-selectin. Ultimately, this may lead to the development of novel EPC-derived antithrombotic therapies in patients with cardiovascular diseases.

## Abbreviations

EPCs: Endothelial progenitor cells
P-sel^−/−^: P-selectin-deficient
WT: Wild-type
COX: Cyclooxygenase
PGI_2_: Prostacyclin
PBMCs: Peripheral blood mononuclear cells
VEGFR2: Vascular endothelial growth factor receptor 2
NO: Nitric oxide
